# Frequent Manipulation of Resistance Training Variables Promotes Myofibrillar Spacing Changes in Resistance-Trained Individuals

**DOI:** 10.3389/fphys.2021.773995

**Published:** 2021-12-15

**Authors:** Carlton D. Fox, Paulo H. C. Mesquita, Joshua S. Godwin, Vitor Angleri, Felipe Damas, Bradley A. Ruple, Casey L. Sexton, Michael D. Brown, Andreas N. Kavazis, Kaelin C. Young, Carlos Ugrinowitsch, Cleiton A. Libardi, Michael D. Roberts

**Affiliations:** ^1^School of Kinesiology, Auburn University, Auburn, AL, United States; ^2^MUSCULAB, Laboratory of Neuromuscular Adaptations to Resistance Training, Department of Physical Education, Federal University of São Carlos, São Carlos, Brazil; ^3^Department of Cell Biology and Physiology, Edward Via College of Osteopathic Medicine – Auburn Campus, Auburn, AL, United States; ^4^School of Physical Education and Sport, University of São Paulo, São Paulo, Brazil

**Keywords:** myofibrils, myosin, actin, sarcoplasm, muscle fiber

## Abstract

We sought to determine if manipulating resistance training (RT) variables differentially altered the expression of select sarcoplasmic and myofibril proteins as well as myofibrillar spacing in myofibers. Resistance-trained men (*n* = 20; 26 ± 3 years old) trained for 8 weeks where a randomized leg performed either a standard (CON) or variable RT protocol (VAR: manipulation of load, volume, muscle action, and rest intervals at each RT session). A pre-training (PRE) vastus lateralis biopsy was obtained from a randomized single leg, and biopsies were obtained from both legs 96 h following the last training bout. The sarcoplasmic protein pool was assayed for proteins involved in energy metabolism, and the myofibril protein pool was assayed for relative myosin heavy chain (MHC) and actin protein abundances. Sections were also histologically analyzed to obtain myofibril spacing characteristics. VAR resulted in ~12% greater volume load (VL) compared to CON (*p* < 0.001). The mean fiber cross-sectional area increased following both RT protocols [CON: 14.6% (775.5 μm^2^), *p* = 0.006; VAR: 13.9% (743.2 μm^2^), *p* = 0.01 vs. PRE for both], but without significant differences between protocols (*p* = 0.79). Neither RT protocol affected a majority of assayed proteins related to energy metabolism, but both training protocols increased hexokinase 2 protein levels and decreased a mitochondrial beta-oxidation marker (VLCAD protein; *p* < 0.05). Citrate synthase activity levels increased with CON RT (*p* < 0.05), but not VAR RT. The relative abundance of MHC (summed isoforms) decreased with both training protocols (*p* < 0.05). However, the relative abundance of actin protein (summed isoforms) decreased with VAR only (13.5 and 9.0%, respectively; *p* < 0.05). A decrease in percent area occupied by myofibrils was observed from PRE to VAR (−4.87%; *p* = 0.048), but not for the CON (4.53%; *p* = 0.979). In contrast, there was an increase in percent area occupied by non-contractile space from PRE to VAR (10.14%; *p* = 0.048), but not PRE to CON (0.72%; *p* = 0.979). In conclusion, while both RT protocols increased muscle fiber hypertrophy, a higher volume-load where RT variables were frequently manipulated increased non-contractile spacing in resistance-trained individuals.

## Introduction

Resistance training (RT) is an effective strategy to increase skeletal muscle fiber cross-section area (fCSA; Kraemer and Ratamess, 2004; American College of Sports Medicine, 2009; Haun et al., 2019b). The frequent manipulation of RT variables (e.g., load, sets, muscle action, and rest) is suggested to enhance muscle hypertrophy compared to standard progressive RT in previously-trained individuals (Kraemer and Ratamess, 2004). Nevertheless, we (Barcelos et al., 2018; Damas et al., 2019; Chaves et al., 2020) and others (Mitchell et al., 2012; Morton et al., 2016) have demonstrated that, when resistance exercises are performed to (or close to) concentric muscle failure, the manipulation of RT variables induces similar increases in fCSA compared to standard RT despite producing a higher volume-load (sets × repetitions × load in kg).

Although muscle fiber hypertrophy is similar when exercising to concentric muscle failure, the differential expansion of the intracellular components (e.g., myofibrillar and sarcoplasmic fractions) and the differential expression of sarcoplasmic proteins may be influenced by different RT protocols. In this regard, MacDougall et al. (1982) first demonstrated that individuals with different RT backgrounds exhibited different myofibrillar and sarcoplasmic spacing characteristics. Recently, we demonstrated that progressively higher RT volume (up to 32 sets of 10 repetitions per muscle group per week) reduced the relative abundance (per milligram of dry tissue) of MHC (combined isoforms) and actin (combined isoforms) by ∼30% in vastus lateralis muscle of resistance-trained young men (Haun et al., 2019a). These findings were further validated with actin-phalloidin staining, which showed decreases in actin density per muscle fiber with this training style. Interestingly, these same participants exhibited a 60% significant increase in sarcoplasmic protein concentrations. Analysis of the sarcoplasmic protein pool from pre- to post-training using shotgun proteomics further indicated that ~40 proteins, most of which were associated with anaerobic and aerobic ATP production [e.g., creatine kinase M-type (CKM), lactate dehydrogenase A (LDHA), phosphofructokinase (PFK), mitochondrial proteins, etc.], were upregulated. In a subsequent study with a separate cohort of resistance-trained men, we demonstrated that a higher load and lower volume RT protocol [lifts ~80–90% of one repetition maximum (1-RM)] relative to that used by Haun et al. increased type II fCSA and reduced the relative abundances of MHC and actin protein (per milligram dry tissue) by only ~3% (Vann et al., 2020a). Additionally, only 12 sarcoplasmic proteins were differentially expressed following training and none of them were involved with energy metabolism (e.g., ATP-PCr, glycolysis, TCA cycle, or beta-oxidation). These findings have led us to hypothesize that the manipulation of RT variables may lead to differential adaptations in the myofibrillar and sarcoplasmic protein pools as well as differential spacing of myofibrils during myofiber hypertrophy (Roberts et al., 2020a). However, no study to date has compared the effects of manipulating RT variables on these aforementioned markers.

Thus, the aim of this study was to investigate whether the manipulation of RT variables in resistance-trained men differentially altered sarcoplasmic and myofibrillar spacing. Additionally, we sought to examine if the expression of sarcoplasmic and myofibril proteins were differentially affected by the manipulation of RT variables. To address these aims, we used unilateral RT protocol where one leg performed a standard progressive RT protocol (CON), and the contralateral leg performed a VAR consisting of daily variation in exercise load, volume, muscle action, and inter-set rest intervals. We hypothesized that, although the CON and VAR would show similar muscle fiber hypertrophy, the space occupied by myofibrils would decrease more so following VAR than CON training due to a higher training volume. Additionally, we hypothesized that VAR protocol would alter the expression of select sarcoplasmic proteins related to energy metabolism.

## Materials and Methods

### Participants and Ethical Approval

The Human Research Ethics Committee of the Federal University of Sao Carlos approved this study prior to data collection (no. 2.226.596). Experimental procedures and associated risks were explained to each participant, who provided written and informed consent before participation. All procedures performed herein were in accordance with the ethical standards of the institutional and national research committee. The subjects herein were the same from our previous study (Damas et al., 2019; *n* = 20, age: 26 ± 3 years, body mass index: 25.6 ± 2.1 kg/m^2^ and previous RT experience: 2.5 ± 1.1 years). As inclusionary criteria, the participants had to be free from musculoskeletal disorders that would prevent adequate performance of the RT protocols, and participants could not be using anabolic steroids. Participants were advised to maintain their eating habits, and to consume a protein supplement provided after every RT session (i.e., 30 g of whey protein isolate).

### General Study Design

**Figure 1:**
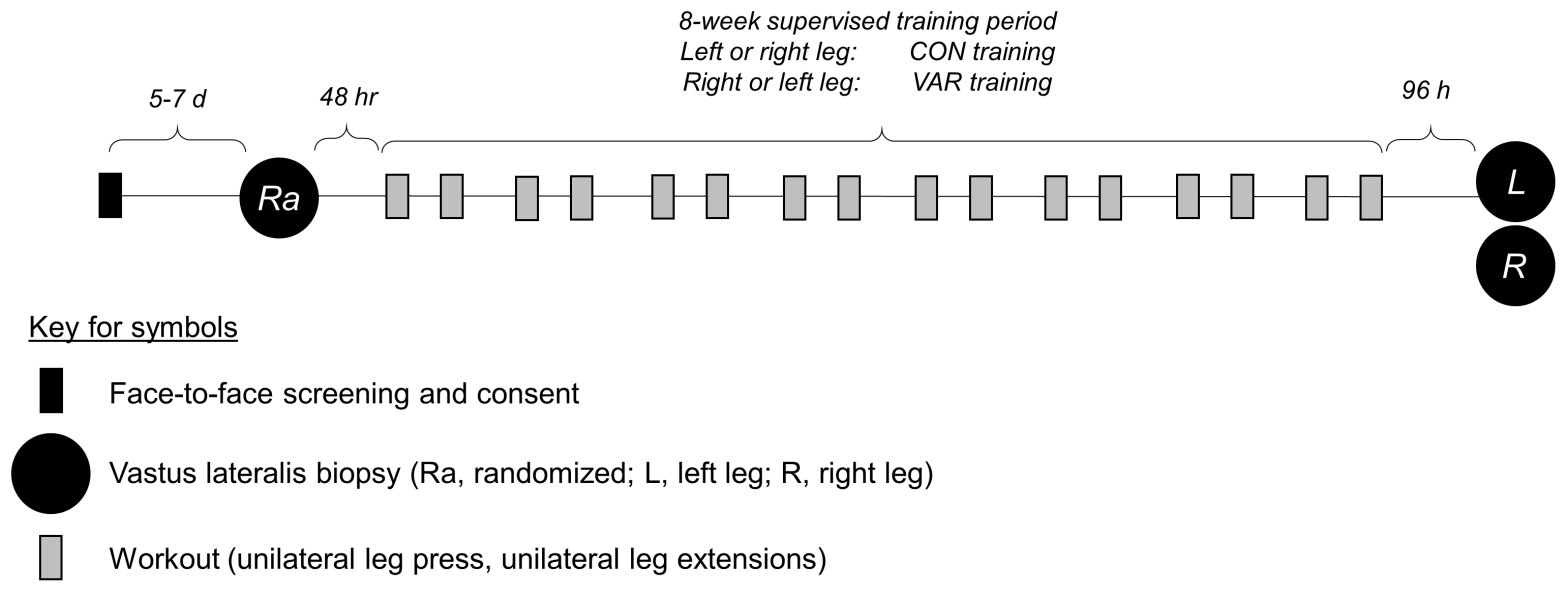
Study design. This shows the study design for the 8-week unilateral leg training paradigm.

All supervised training and tissue procurement occurred at the Federal University of Sao Carlos, and most wet laboratory analyses occurred at Auburn University. For RT, a within-subjects unilateral study design was utilized. The within-subject design is effective in controlling biological variability as between-leg responses are identically affected by genetic, nutritional, fitness, and sleep conditions (Franchi et al., 2015, 2018; MacInnis et al., 2017). All participants completed a familiarization session with all training protocols occurring before the onset of RT. Afterwards, participants performed twice-weekly unilateral leg RT over 8 weeks. One leg was randomly assigned to a standard progressive RT paradigm (CON, 10 dominant and 10 non-dominant legs). The contralateral leg was assigned to a VAR (10 dominant and 10 non-dominant legs). The VAR protocol systematically and sequentially modified exercise-related variables at each RT session (see full descriptions of RT protocols below). Before the 8-week training protocol (PRE), a single muscle biopsy was obtained from the vastus lateralis, where the leg used for collection was randomized (CON, *n* = 10 or VAR, *n* = 10). Bilateral vastus lateralis muscle biopsies were obtained after 8 weeks of CON or VAR training (96 h after the last RT session). A summary figure of the study design is presented below (Figure 1), and more details related to testing and training have been previously published (Damas et al., 2019).

### Resistance Training Protocol

The CON protocol consisted of eight sets (four sets of unilateral leg press followed by four sets of unilateral leg extension exercise) of 9–12 repetitions of resistance exercise to concentric muscle failure with 2-min rest intervals between sets. This repetition range was achieved by increasing or decreasing the load between sets as previously described (Damas et al., 2019). Each session of the VAR protocol involved one of the following RT manipulations, which were performed in a randomized and balanced fashion: (i) variable-load, eight sets (four of leg press and four of leg extensions) of 25–30 reps to concentric failure/2 min between set rest interval; (ii) variable-sets, 12 sets (six of leg press and six of leg extension) of 9–12 reps to concentric failure/2 min between set rest intervals; (iii) variation in contraction type, eight sets (four of leg press and four of leg extension) of 10 eccentric contractions at 110% of the load used in the CON leg/2 min between set rest intervals; and (iv) variations in rest periods, eight sets (four of leg press and four of leg extension) of 9–12 reps to concentric failure/4 min between set rest intervals. The volume-load performed by the CON and VAR legs over the training period was calculated considering all RT sessions.

### Muscle Biopsies

Biopsies of the vastus lateralis were performed using the percutaneous muscle biopsy technique with suction under local anesthesia 2–3 ml of 1% Xylocaine. Approximately 50 mg muscle tissue was dissected free from blood and connective tissue, and muscle tissue samples were immediately placed in cryogenic tubes, frozen in liquid nitrogen, and stored at −80°C until analyses described below. Separate pieces of muscle were preserved slow-frozen in OCT media, sectioned at a thickness of 6 μm using a cryostat, mounted on positively charged microscope slides, and stored at −80°C. Frozen samples and a subset of mounted sections were subsequently shipped to Auburn University on dry ice, and Auburn investigators received samples and stored them at −80°C until analyses described below.

### Muscle Fiber Cross-Section Area

Mean fCSA analysis was the only wet laboratory procedure not performed by the Auburn University group. Frozen muscle samples were sectioned using a cryostat (Leica CM 1860) at −25°C. The slides with muscle cross sections (6 μm) were left at room temperature for ~20 min to stabilize. Samples were incubated with the primary antibodies [anti-Laminin (Abcam – ab11575) and anti-MHCI (DSHB – BA-D5)] at 37°C for 45 min. The samples were washed three times for 5 min in phosphate buffered saline (PBS), and then were incubated with the secondary antibodies [alexa 488 (Abcam – ab150077) and alexa 488 (DSHB – 115-547-187)] at 37°C for 45 min. After three more 5-min washes in PBS, the sections were mounted in fluorescent media. The images were obtained with an ImageXpress Micro XLS with a magnification of 20×. The analyses were performed in the ImageJ software (NIH, Bethesda, MD, United States). fCSA values were determined using computerized planimetry. To ensure that only cross-sectioned fibers were analyzed, fibers with circularity below 0.60 were excluded from the analysis (Verdijk et al., 2009). The mean (range) number of analyzed fibers was 98 (78–100) at PRE, 91 (26–100) at POST (CON), and 96 (78–100) at POST (VAR). The coefficient of variation (CV) between two measurements performed 72 h apart was 2.8%.

### Sarcoplasmic and Myofibrillar Protein Isolations

Isolation of the sarcoplasmic and myofibrillar protein fractions from wet muscle tissue was performed using our recently published “MIST” or “myofibrillar isolation and solubilization technique” (Roberts et al., 2020b). Briefly, 1.7 ml polypropylene tubes were pre-filled with ice-cold buffer (300 μl, Buffer 1: 25 mM Tris, pH 7.2, 0.5% Triton X-100, protease inhibitors) and placed on ice. Skeletal muscle foils were removed from −80°C, placed on a liquid nitrogen-cooled ceramic mortar and pestle, and tissue was pulverized into 2–4 mm^3^ pieces. Approximately ~20 mg of crushed tissue was placed in tubes prefilled with buffer (described above), weighed using a scale with a sensitivity of 0.0001 g (Mettler-Toledo; Columbus, OH, United States), and placed on ice. Samples were homogenized using tight-fitting pestles and centrifuged at 1,500 *g* for 10 min at 4°C. Supernatants (sarcoplasmic fraction) were collected and placed in new 1.7 ml polypropylene tubes on ice. As a wash step, the resultant myofibrillar pellet was resuspended in 300 μl of Buffer 1 and centrifuged at 1,500 *g* for 10 min at 4°C. The supernatant was discarded and the myofibrillar pellet was solubilized in 300 μl of ice-cold resuspension buffer (20 mM Tris-HCl, pH 7.2, 100 mM KCl, 20% glycerol, 1 mM DTT, 50 mM spermidine, protease inhibitors). Protein concentrations for the sarcoplasmic fraction were determined the same day as protein isolations to minimize freeze-thaw effects, and the myofibrillar fraction was prepared for actin and MHC protein abundance (described below), and stored at −80°C until analysis occurred.

### Determination of Protein Concentration

Sarcoplasmic protein resuspensions were batch-assayed for determination of protein concentration using a commercially available bicinchoninic acid kit (Thermo Fisher Scientific; Waltham, MA, United States). Samples were assayed in duplicate using a microplate assay protocol where a small volume of sample was assayed (20 μl of 5× diluted sample + 200 μl Reagent A + B). The average duplicate CV for sarcoplasmic protein concentrations were 9.1%.

### SDS-PAGE and Coomassie Staining for Relative Contractile Protein Abundance

Determination of contractile protein abundances per mg wet tissue were performed as previously described by our laboratory and others (Dowling et al., 2019; Haun et al., 2019a). Briefly, SDS-PAGE sample preps were made using 10 μl resuspended myofibrils, 65 μl distilled water (diH_2_O), and 25 μl 4× Laemmli buffer. Samples (5 μl) were then loaded on precast gradients (4–15%) SDS-polyacrylamide gels (Bio-Rad Laboratories; Hercules, CA, United States) and subjected to electrophoresis at 180 V for 40 min using pre-made 1× SDS-PAGE running buffer (VWR International; Randor, PA, United States). Following electrophoresis, gels were rinsed in diH_2_O for 15 min and immersed in Coomassie stain (LabSafe GEL Blue; G-Biosciences; St. Louis, MO, United States) for 2 h. Gels were then destained in diH_2_O for 60 min, and band densitometry was performed using a gel documentation system and associated software (ChemiDoc; Bio-Rad Laboratories). Given that a standardized volume from all samples was loaded onto gels, MHC and actin band densities were normalized to wet muscle weight to derive relative protein abundances expressed as arbitrary density units (ADU) per mg wet muscle. Our laboratory has reported that this method yields good sensitivity in detecting 5–25% increases in relative MHC and actin protein abundances (Roberts et al., 2018).

### Western Blotting of Sarcoplasmic Protein Fraction

Sarcoplasmic protein resuspensions obtained above were prepared for Western blotting at 1 μg/μl using 4× Laemmli buffer. Following sample preparation, 15 μl samples were loaded onto pre-casted gradient (4–15%) SDS-polyacrylamide gels (Bio-Rad Laboratories) and subjected to electrophoresis (180 V for 45–60 min) using pre-made 1× SDS-PAGE running buffer (VWR International). Proteins were subsequently transferred (200 mA for 2 h) to polyvinylidene difluoride membranes (PVDF; Bio-Rad Laboratories), Ponceau stained, and imaged to ensure equal protein loading between lanes. Membranes were blocked for 1 h at room temperature with 5% nonfat milk powder in Tris-buffered saline with 0.1% Tween-20 (VWR International). Membranes were then incubated overnight at 4°C with the following antibody cocktails (each at a 1:1,000 dilution) in TBST with 5% bovine serum albumin (BSA): (i) goat anti-human creatine kinase (CKM; Abcam; Cambridge, MA, United States; cat# ab174672), (ii) rabbit anti-human L-type amino acid transporter (LAT1, Cell Signaling Technology; Danvers, MA, United States; cat# 5347), (iii) rabbit anti-human glucose transporter 4 (GLUT4; Cell Signaling Technology, cat# 2213), (iv) rabbit anti-human lactate dehydrogenase (LDHA; Cell Signaling Technology, cat# 2012), (v) rabbit anti-human phosphofructokinase (PFKM; Abcam, cat#: ab154804), (vi) rabbit anti-human total oxidative phosphorylation (OXPHOS) cocktail (Abcam, cat# ab110411), (vii) rabbit anti-human complex IV (Cell Signaling Technology, cat# 4850), (viii) rabbit anti-human isocitrate dehydrogenase 2 (IDH2; Cell Signaling Technology, cat# 56439), (ix) rabbit anti-human acyl-CoA dehydrogenase very long chain (VLCAD; Abcam, cat# ab188872), (x) rabbit anti-human carnitine palmitoyltransferase I (CPTI; Abcam, cat# ab134135), and (xi) rabbit anti-human hexokinase II (HK2; Cell Signaling Technology, cat# 2867). The following day, membranes were incubated with horseradish peroxidase (HRP)-conjugated anti-rabbit IgG (Cell Signaling, cat#7074), or HRP-conjugated anti-goat IgG (GeneTex, cat# GTX628547-01) in TBST with 5% BSA at room temperature for 1 h (secondary antibodies diluted 1:2,000). Membrane development was performed using an enhanced chemiluminescent reagent (Luminata Forte HRP substrate; EMD Millipore, Billerica, MA, United States), and band densitometry was performed using a gel documentation system and associated software (Bio-Rad Laboratories). Raw densitometry values for each target were divided by whole-lane Ponceau densities. All values were then divided by the mean of the PRE time point to depict relative protein concentrations.

### Citrate Synthase Activity Assay

Sarcoplasmic protein resuspensions obtained above were prepared for citrate synthase (CS) activity assays as previously described by our laboratory (Haun et al., 2019a). This metric was used as a surrogate for mitochondrial content per the findings of Larsen et al. (2012), suggesting who suggest CS activity correlates with transmission electron micrograph images of mitochondrial content. The assay principle is based upon the reduction of 5,50-dithiobis (2-nitrobenzoic acid; DTNB) at 412 nm (extinction coefficient 13.6 mmol/L/cm) coupled to the reduction of acetyl-CoA by the CS reaction in the presence of oxaloacetate. Lysates from each biopsy (providing 12.5 μg protein) were assayed in duplicate where samples were added to a mixture composed of 0.125 mol/L Tris–HCl (pH 8.0), 0.03 mmol/L acetyl-CoA, and 0.1 mmol/L DTNB. The reaction was initiated by adding 5 μl of 50 mmol/L oxaloacetate and the absorbance change was recorded for 1 min. The CV for all duplicates was 5.7%.

### Phalloidin Staining

For the determination of myofibril area per fiber, F-actin labelling using Alexa Fluor 488-conjugated (AF488) phalloidin was performed according to previous reports (Gokhin et al., 2008; Duddy et al., 2015; Haun et al., 2019b). Critically, we recently utilized this method (Ruple et al., 2021) and showed that estimates of intracellular area occupied by myofibrils (~75–80%) agree with estimates provided by transmission electron microscopy (TEM) studies (MacDougall et al., 1982; Alway et al., 1988), which has been viewed as the gold standard when examining adaptations intramyocellular to RT. Briefly, serial sections were air-dried for 10 min followed by 10% formalin fixation for 10 min. Sections were then washed with PBS for 5 min, and blocked with 100% Pierce Super Blocker (Thermo Fisher Scientific) for 25 min. After blocking, a pre-diluted commercially-available rabbit anti-dystrophin IgG1 antibody solution (Genetex, cat# GTX15277) and spiked in phalloidin-AF488 (1:50 dilution) was placed on sections in the dark for 30 min. Sections were subsequently washed in PBS for 5 min and incubated in the dark for 30 min with a secondary antibody solution containing Texas Red-conjugated anti-rabbit IgG (Vector Laboratories, cat# TI-1000; ~6.6 μl secondary antibody per 1 ml of blocking solution). Sections were washed in PBS for 5 min, and air-dried and mounted with fluorescent media containing DAPI (Genetex, cat# GTX16206). Following mounting, digital images were immediately captured with a fluorescent microscope (Nikon Instruments; Melville, NY, United States) using a 20× objective. Exposure times were 200 ms for FITC, 800 ms for TRITC imaging, and 100 ms for DAPI imaging. This staining method allowed the identification of the sarcolemma (Texas Red filter), myofibrils (FITC filter), and myonuclei (DAPI filter). ImageJ (NIH) was used to quantify myofibril area per fiber. Briefly, images were split into RGB channels, and the green channel image was converted to grayscale. The threshold function in imageJ was then used to generate binary black/white images of stained vs. unstained portions of fibers. Thereafter, fibers were manually traced using the polygon function, and myofibril areas were provided as a percentage per fiber area. A visual representation of this image analysis is provided in the results section.

### Statistical Analysis

All statistics were performed using GraphPad Prism 6.0 (GraphPad Software, Inc., San Diego, CA). As PRE data analyses were performed based on the muscle tissue harvested from just one leg, we used one-way ANOVAs to determine if the dependent variables (fCSA, protein expression and histology characteristics) at post (CON and VAR) were different from PRE. Dunnett’s *post-hoc* tests were performed, in case of significant *F*-values, having PRE as the control condition (Howell, 2013). Change score values of each protocol (PRE to CON and PRE to VAR) were also compared using paired t-tests. Select associations were also performed using Pearson’s correlations. Statistical significance for null hypothesis testing was set at *p* < 0.05. Data are presented throughout as mean ± standard deviation values.

## Results

### Volume-Load and Muscle Fiber Cross-Section Area

Variable RT protocol showed significantly greater volume-load values (217,613 ± 43,834 kg) compared with CON (193,259 ± 39,731 kg, 12.6% difference between legs; *p* < 0.001; *results not shown in figure*). Mean fCSA increased significantly from PRE (5,994 ± 1,000 μm^2^) to CON (6,770 ± 1,000 μm^2^, *p* = 0.006) and VAR (6,738 ± 1,070 μm^2^, *p* = 0.01; *results not shown in figure*). No significant difference was found between change scores of each protocol (CON: 775.5 ± 997.0 μm^2^ and VAR: 743.2 ± 1,023.0 μm^2^; *p* = 0.791).

### Sarcoplasmic Enzyme Proteins

**Figure 2:**
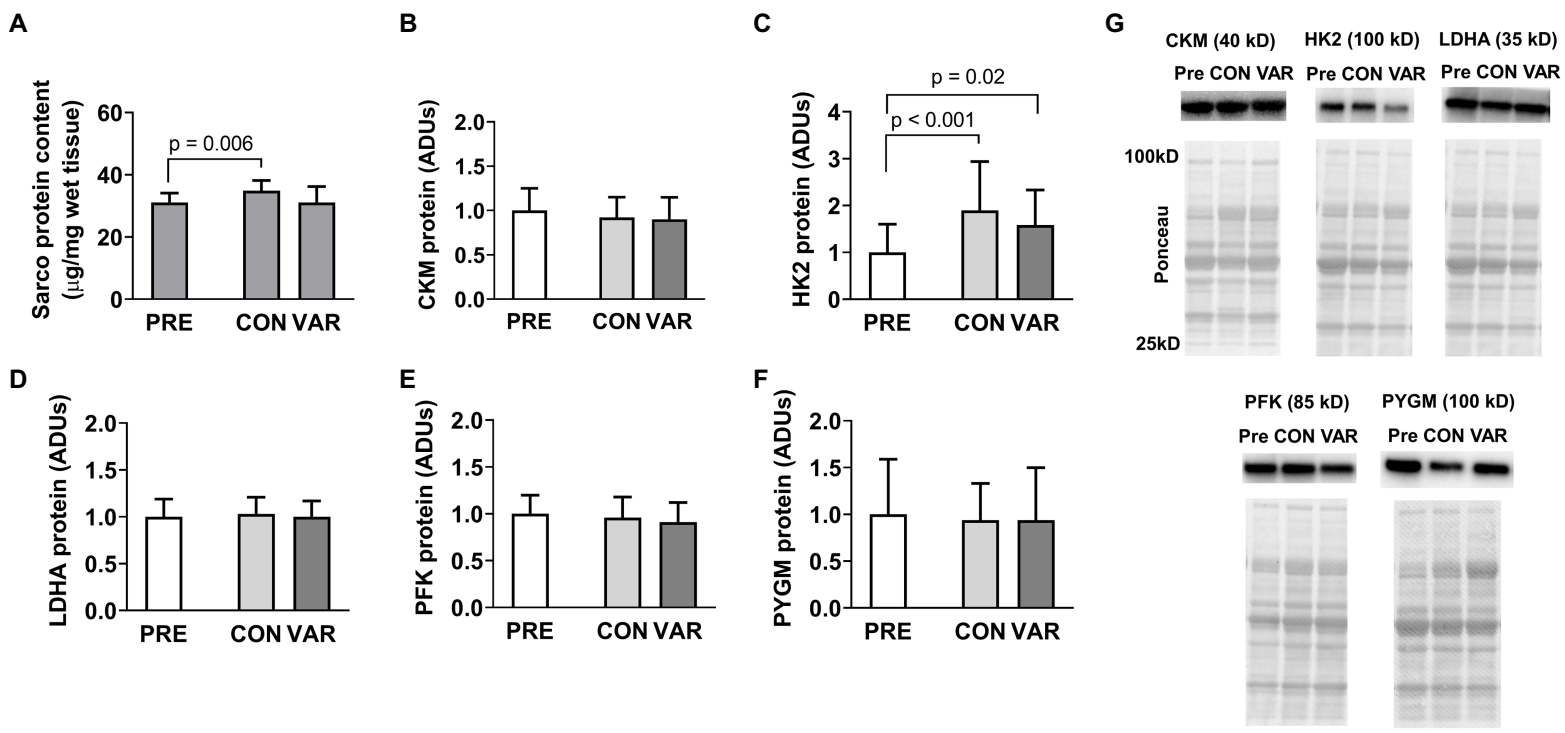
Alterations in select sarcoplasmic enzymes with CON vs. VAR training. Data presented in arbitrary units (ADUs) include sarcoplasmic protein concentrations from biopsied muscle (panel **A**), creatine kinase, M-type (CKM) protein levels from the sarcoplasmic protein pool (panel **B**), hexokinase 2 (HK2) protein levels from the sarcoplasmic protein pool (panel **C**), lactate dehydrogenase A (LDHA) protein levels from the sarcoplasmic protein pool (panel **D**), phosphofructokinase (PFK) protein levels from the sarcoplasmic protein pool (panel **E**), and glycogen phosphorylase (PYGM) protein levels from the sarcoplasmic protein pool (panel **F**). Data are presented as means with standard deviation bars. Panel **(G)** contains representative Western blot images for each target. Note that two of Ponceau images in panel **(G)** (for HK2 and LDHA) are the same given that the same subject was used for the representative Western blot images of these targets.

Changes in sarcoplasmic enzyme protein content are presented in Figure 2. Total sarcoplasmic protein content increased significantly from PRE (31.05 ± 2.98 μg/mg) to CON (34.89 μg/mg; *p* = 0.006), but not for the VAR (31.07 ± 5.09 μg/mg; *p* > 0.99; Figure 2A). There was also a difference between delta of change of CON (3.83 ± 2.98 μg/mg) and VAR (0.01 ± 5.85 μg/mg; *p* = 0.046). Additionally, we observed a significant change in HK2 protein levels from PRE [1.00 ± 0.59 arbitrary units (ADUs)] to CON (1.90 ± 1.03 ADUs; *p* < 0.001) and VAR (1.58 ± 0.74 ADUs; *p* = 0.02; Figure 2C). However, no significant difference was observed between the change scores of the training protocols (CON: 0.89 ± 0.82 ADUs and VAR: 0.57 ± 0.94 ADUs; *p* = 0.229). CKM (Figure 2B), LDHA (Figure 2D), PFK (Figure 2E), and PYGM (Figure 2F) protein levels demonstrated no differences from PRE (1.00 ± 0.24 ADUs, 1.00 ± 0.19 ADUs, 1.00 ± 0.20 ADUs, 1.00 ± 0.59 ADUs, respectively) to CON (0.92 ± 0.22 ADUs, 1.02 ± 0.18 ADUs, 0.96 ± 0.21 ADUs, 0.94 ± 0.39 ADUs, respectively; *p* > 0.05) or VAR (0.89 ± 0.25 ADUs, 0.99 ± 0.17 ADUs, 0.91 ± 0.20 ADUs, 0.93 ± 0.55 ADUs, respectively; *p* > 0.05). Additionally, no significant difference was observed between change scores of the CON and VAR protocols (CKM: −0.07 ± 0.20 ADUs and −0.10 ± 0.24 ADUs, respectively; LDHA: 0.02 ± 0.15 ADUs and −0.00005 ± 0.16 ADUs, respectively; PFK: −0.03 ± 0.12 ADUs and −0.08 ± 0.17 ADUs, respectively; PYGM: −0.05 ± 0.44 ADUs and −0.06 ± 0.94 ADUs, respectively; *p* > 0.05). Figure 2G provides representative Western blot images for assayed proteins.

### Select Nutrient Transporter Protein Levels

**Figure 3:**
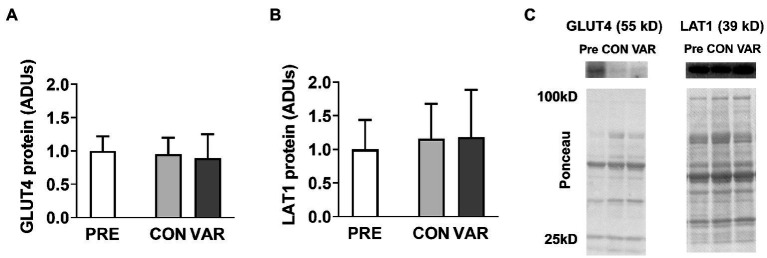
Alterations in select nutrient transporters with CON vs. VAR training. Data presented in arbitrary units (ADUs) include glucose transporter 4 (GLUT4) protein levels from the sarcoplasmic protein pool (panel **A**), and L-Type Amino Acid Transporter 1 (LAT1) protein levels from the sarcoplasmic protein pool (panel **B**). Data are presented as means with standard deviation bars. Panel **(C)** contains representative Western blot images for each target.

Select nutrient transporter protein content changes are presented in Figure 3. No significant differences were found from PRE (1.00 ± 1.22 ADUs) to CON (0.95 ± 0.24 ADUs; *p* = 0.979) or VAR (0.88 ± 0.36 ADUs; *p* = 0.903) for GLUT4 (Figure 3A). Similarly, no significant differences were observed from PRE (1.00 ± 0.43 ADUs) to CON (1.15 ± 0.51 ADUs; *p* = 0.304) and VAR (1.18 ± 0.70 ADUs; *p* = 0.398) for LAT1 (Figure 3B). No significant differences were found between the change scores of the CON and VAR protocols (GLUT4: −0.04 ± 1.23 ADUs and −0.11 ± 1.33 ADUs, respectively; LAT1: 0.15 ± 0.50 ADUs and 0.18 ± 0.60 ADUs, respectively; *p* > 0.05). Figure 3C provides representative Western blot images for assayed proteins.

### Mitochondrial Enzyme Markers

**Figure 4:**
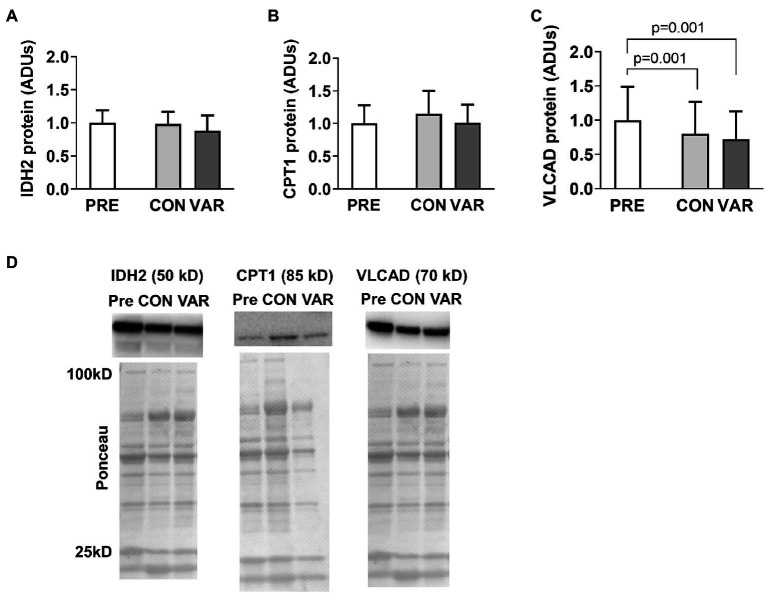
Alterations in select mitochondrial enzymes with CON vs. VAR training. Data presented in arbitrary units (ADUs) include isocitrate dehydrogenase 2 (IDH2) protein levels from the sarcoplasmic protein pool (panel **A**), carnitine palmitoyltransferase 1 (CPT1) protein levels from the sarcoplasmic protein pool (panel **B**), and very long-chain specific acyl-CoA dehydrogenase (VLCAD) protein levels from the sarcoplasmic protein pool (panel **C**). Data are presented as means with standard deviation bars. Panel **(D)** contains representative Western blot images for each target. Note that two of Ponceau images in panel **(D)** (for IDH2 and VLCAD) are the same given that the same subject was used for the representative Western blot images of these targets.

The protein content of select mitochondrial enzymes is presented in Figure 4. No significant differences were found from PRE (1.00 ± 0.18 ADUs) to CON (0.98 ± 0.18 ADUs; *p* = 0.874) or VAR (0.88 ± 0.22 ADUs; *p* = 0.142) for IDH2 (Figure 4A). Similarly, no significant differences were observed from PRE (1.00 ± 0.28 ADUs) to CON (1.14 ± 0.35 ADUs; *p* = 0.192) or VAR (1.00 ± 0.27 ADUs; *p* = 0.995) for CPT1 (Figure 4B). No significant differences were found between the change scores of the CON and VAR protocols (IDH2: −0.01 ± 0.20 ADUs and −0.11 ± 0.28 ADUs, respectively; CPT1: 0.14 ± 0.40 ADUs and 0.006 ± 0.34 ADUs, respectively; *p* > 0.05). For VLCAD protein levels, CON and VAR were lower (0.79 ± 0.47 ADUs and 0.72 ± 0.40 ADUs, respectively) compared to PRE values (1.00 ± 0.48 ADUs; *p* = 0.001 for both), albeit no significant differences were found between the change scores of the CON and VAR protocols (−0.21 ± 0.23 ADUs and −0.28 ± 0.32 ADUs, respectively; *p* = 0.338; Figure 4C). Figure 4D provides representative Western blot images for assayed proteins.

### Mitochondrial Markers

**Figure 5:**
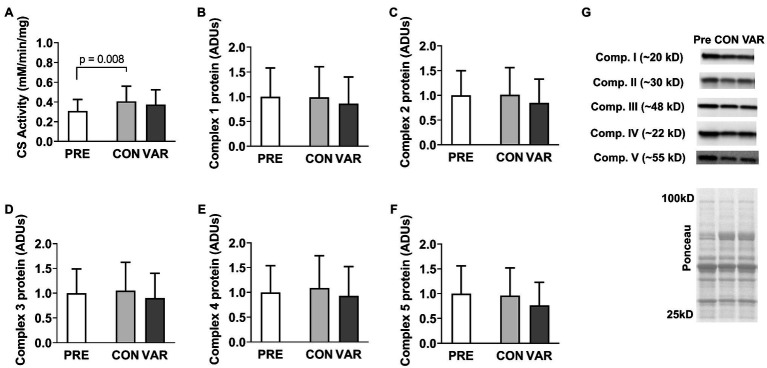
Alterations in citrate synthase activity levels and mitochondrial electron transport chain proteins with CON vs. VAR training. Data presented include citrate synthase (CS) activity levels from the sarcoplasmic protein pool (panel **A**), and the protein levels of complexes 1–5 from the sarcoplasmic protein pool (panels **B–F**). Data are presented as means with standard deviation bars. Panel **(G)** contains representative Western blot images for each target. Arbitrary units (ADUs).

Citrate synthase activity and protein content for mitochondrial respiration complexes I–V are presented in Figure 5. CS activity increased from PRE (0.30 ± 0.11 mM/min/mg) to CON (0.40 ± 0.15 mM/min/mg, *p* = 0.008), but not PRE to VAR (0.37 ± 0.15 mM/min/mg, *p* = 0.192; Figure 5A), although no difference in CS activity was found between the change scores of the CON and VAR protocols (0.10 ± 0.13 mM/min/mg and 0.06 ± 0.11 mM/min/mg, respectively; *p* = 0.250). Complexes I/II/III/IV/V did not show significant changes from PRE (1.00 ± 0.58 ADUs, 1.00 ± 0.49 ADUs, 1.00 ± 0.48 ADUs, 1.00 ± 0.53 ADUs, 1.00 ± 0.55 ADUs, respectively) to CON (0.98 ± 0.61 ADUs, 1.00 ± 0.54 ADUs, 1.04 ± 0.56 ADUs, 1.09 ± 0.64 ADUs, 0.96 ± 0.56 ADUs, respectively) and VAR (0.85 ± 0.53 ADUs, 0.84 ± 0.47 ADUs, 0.90 ± 0.49 ADUs, 0.93 ± 0.58 ADUs, 0.76 ± 0.46 ADUs, respectively; *p* > 0.05; Figures 5B-F). Additionally, no significant differences were found between the change scores of the CON and VAR protocols (Complex 1: −0.01 ± 0.26 ADUs and −0.14 ± 0.53 ADUs, respectively; Complex 2: 0.01 ± 0.23 ADUs and −0.15 ± 0.36 ADUs, respectively; Complex 3: 0.04 ± 0.24 ADUs and −0.09 ± 0.35 ADUs, respectively; Complex 4: 0.10 ± 0.33 ADUs and −0.05 ± 0.46 ADUs, respectively; Complex 5: −0.03 ± 0.30 ADUs and −0.23 ± 0.48 ADUs, respectively; *p* > 0.05). Figure 5G provides representative Western blot images for assayed proteins.

### Contractile Protein Markers

**Figure 6:**
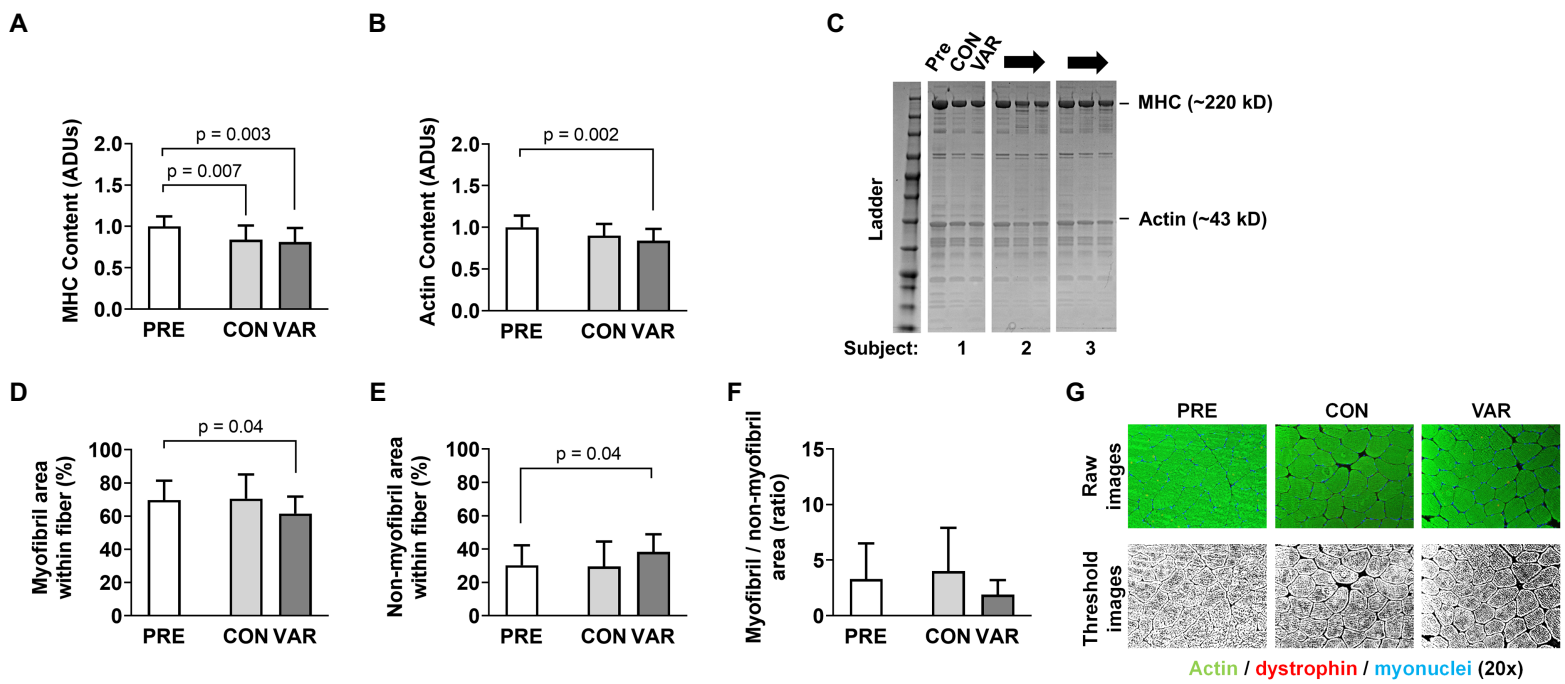
Effect of CON and VAR training on contractile protein markers. Data presented include the relative abundances of myosin heavy (MHC) and actin protein levels from the myofibril protein pool (panels **A,B**) as determined by SDS-PAGE and Coomassie staining. Data in panels **(D–F)** were derived from phalloidin staining and image analysis. Data are presented as means with standard deviation bars. Panels **(C,G)** contain representative images of SDS-PAGE and phallodin staining, respectively. Arbitrary units (ADUs).

**Figure 7:**
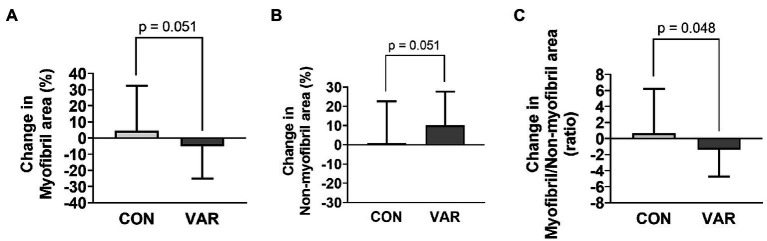
Changes in contractile protein markers with CON vs. VAR training. Data presented include the changes in myofibril (panel **A**), non-myofibril area (panel **B**), and myofibril/non-miofibril ratio (panel **C**).

Changes in contractile protein markers are presented in Figure 6. The relative abundance of MHC (per mg wet tissue) decreased from PRE (1.00 ± 0.11 ADUs) to CON (0.83 ± 0.17 ADUs; *p* = 0.007) and VAR (0.80 ± 0.17 ADUs; *p* = 0.003; Figure 6A). However, no differences were observed between the change scores of CON and VAR protocols (−0.16 ± 0.21 ADUs and −0.19 ± 0.22 ADUs, respectively; *p* = 0.635). A significant decrease was also observed for the relative abundance of actin protein (per mg wet tissue) from PRE (1.00 ± 0.13 ADUs) to VAR (0.84 ± 0.14 ADUs; *p* = 0.004), but not for the CON (0.89 ± 0.14 ADUs; Figure 6B). However, no differences were observed between of the change scores of the CON and VAR protocols (−0.10 ± 0.21 ADUs and −0.15 ± 0.19 ADUs, respectively; *p* = 0.137). A decrease in percent area occupied by myofibrils was observed from PRE (69.73 ± 12.01%) to VAR (61.60 ± 10.52%; *p* = 0.048), but not for the CON (70.55 ± 61.59%; *p* = 0.979). In contrast, there was an increase in percent area occupied by non-contractile space from PRE (30.27 ± 12.01%) to VAR (38.42 ± 10.52%; *p* = 0.048), but not PRE to CON (29.46 ± 14.98%; *p* = 0.979; Figures 6D,E). However, despite the strong trend, no significant differences were found between the change scores of the CON and VAR protocols for the space occupied by myofibrillar (4.53 ± 27.90% and −4.87 ± 20.15%; *p* = 0.051) and non-contractile proteins (0.72 ± 22.00% and 10.14 ± 17.48%; *p* = 0.051; Figures 7A,B). Finally, when considering the ratio of space occupied by myofibrils vs. the space occupied by non-myofibrils, no significant differences were found from PRE (3.31 ± 3.15 ratio) to CON (3.96 ± 3.90 ratio; *p* > 0.05) or VAR (1.92 ± 1.24 ratio; *p* > 0.05; Figure 6F). However, the change score of the VAR protocol was greater than the CON protocol (−1.39 ± 3.32 ratio and 0.64 ± 5.54 ratio; *p* = 0.048; Figure 7C). Figure 6G provides representative histology images for actin-phalloidin staining.

### Contractile Protein Correlations With VAR Training

**Figure 8:**
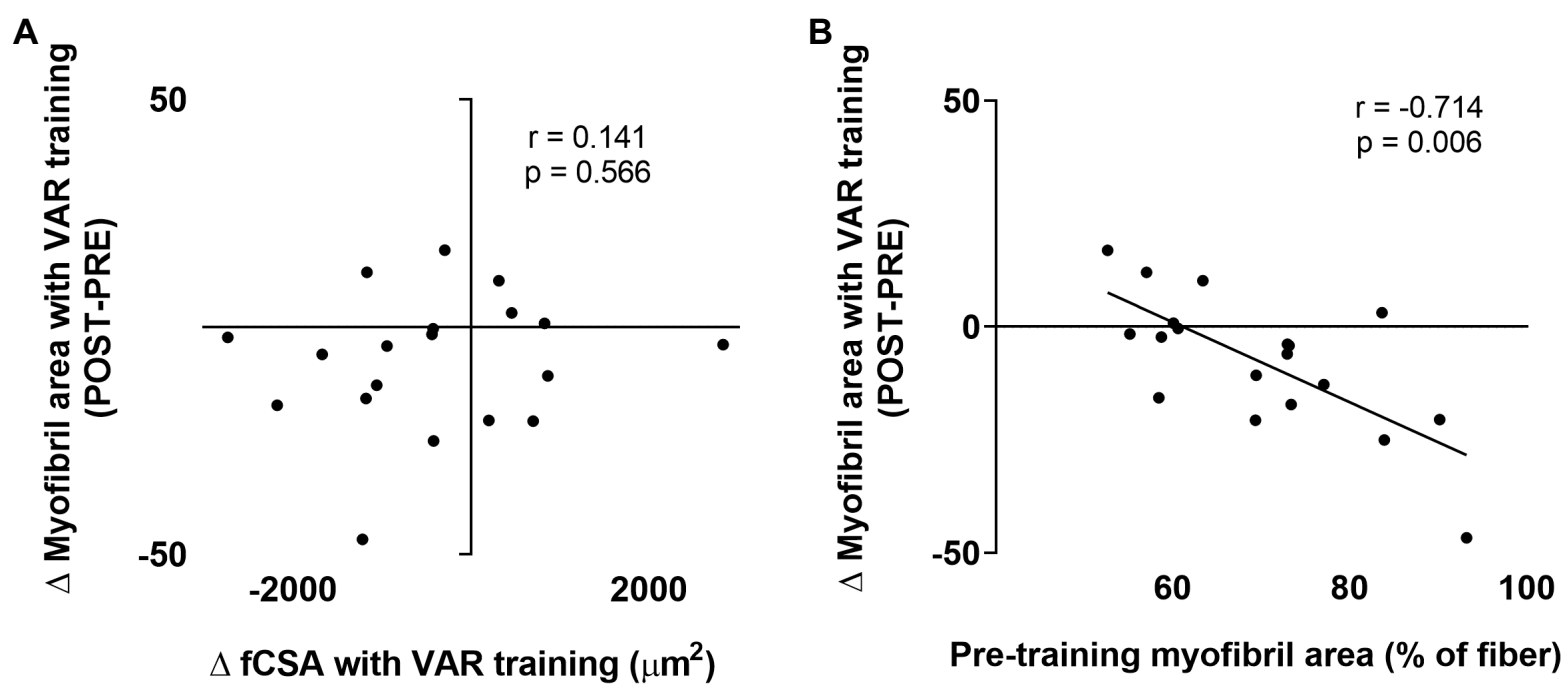
Select contractile protein marker correlations with VAR training. The first association is the pre-to-post training change in mean fCSA with VAR training vs. the change in myofibril area (panel **A**); no significant association was evident. The second is the pre-training myofibril area vs. the change in myofibril area with VAR training (panel **B**); a significant negative association was evident.

Given that we observed a decrease in myofibril area with VAR training, we were interested in select associations with this variable. No association was observed between the changes in fCSA with VAR training vs. the changes in myofibril area (Figure 8A). However, a significant negative association was observed between pre-training myofibril area within muscle fibers vs. the VAR-induced change in myofibril area (*r* = −0.714, *p* = 0.006; Figure 8B).

## Discussion

We demonstrated that frequent manipulation of RT variables does not affect several proteins in the sarcoplasmic fraction such as enzymes related to energy production (CKM, LDHA, PFK, and PYGM), nutrient transporters (LAT1 and GLUT4), mitochondrial enzymes (IDH2 and CPT1), or markers of mitochondrial volume (COX I, COX II, COX III, COX IV, and COX V proteins, except the CS activity which increased for CON). However, we observed that the total sarcoplasmic protein concentrations significantly increased following CON, and CON values were also greater than the VAR after the training period. In addition, HK2 protein increased in both protocols, and very long chain Acyl CoA dehydrogenase (VLCAD) decreased in both protocols. Perhaps most interesting, the VAR protocol decreased the intracellular area occupied by myofibrils, despite increasing muscle fCSA similar to the CON protocol. Moreover, individuals that began training with more myofibril packing (i.e., less non-contractile spacing) experienced a greater increase in non-myofibril space following VAR.

### Sarcoplasmic Protein Concentrations

As mentioned above, the total sarcoplasmic protein concentrations increased only for the CON protocol. However, this increase was not accompanied by changes in CKM, PFK, PYGM, or LDHA protein levels, with the exception HK2 protein content, which increased with training regardless of the protocol. Collectively, these markers were chosen to reflect the ATP-PCr and glycolytic pathways given that we have adopted a similar previous approach using proteomics and downstream bioinformatics (Vann et al., 2020b). In lieu of some of our laboratory’s previous findings, we posited that higher volume RT might largely facilitate metabolic adaptation in muscle tissue, whereas lower volume RT does not, although changes in sarcoplasmic proteins associated with ATP production are not always aligned with changes in total sarcoplasmic protein content. However, contrary to our hypothesis, this was not the case. It is difficult to compare the current study to these prior studies given that exercise selections, modes of training (e.g., bilateral training for the prior studies vs. unilateral training herein), and training durations differed. In the present study, the VAR resulted in a greater volume-load (VL; 217,613 kg) compared to the CON (193,259 kg). Participants in the Haun et al. (2019a) study, who showed signs of metabolic adaptation, were subjected to very high training volumes where the VL for lower body exercises was 206,260 kg on average. In contrast, participants in the Vann et al. (2020a) study, who showed no signs of metabolic adaptation, logged an average lower body VL of 101,820 kg on average. Given the collective evidence, we posit that, although the VAR leg experienced a frequent variation in RT and a VL ~12% greater than the CON leg, both legs likely did not undergo a volume-load stimulus that was enough to induce changes in the assayed markers related to energy metabolism.

### Nutrient Transporters

Beyond interrogating sarcoplasmic proteins related to metabolic adaptation, we investigated the effects of manipulating RT variables on select nutrient transporters (LAT1 and GLUT4). LAT1 transports L-leucine and other essential amino acids into myofibers through a bi-transport system that simultaneously exports glutamine. This transporter is critical for muscle physiology for two reasons. First, leucine stimulates the mechanistic target of rapamycin complex 1 (mTORC1) to increase muscle protein synthesis (Hodson et al., 2017). Second, skeletal muscle can catabolize branched chain amino acids (BCAAs) during higher-volume endurance exercise to produce ATP (Neinast et al., 2019). In the present study, we did not observe changes in LAT1 protein levels for either 8-week RT protocol. In contrast, we recently reported that skeletal muscle LAT1 protein levels increased in untrained subjects following 12 weeks of RT (Roberson et al., 2021). To our knowledge, this is the first study to investigate the chronic effects of RT on LAT1 in resistance-trained individuals. It is notable that others have reported resistance-trained individuals do not show increases in LAT1 protein expression after a single RT session (Hannaian et al., 2020). Collectively, these findings suggest that resistance-trained individuals may be less sensitive to changes in LAT1 protein levels compared to untrained participants. Future studies comparing trained and untrained individuals can contribute to a better understanding of the effects of RT on this nutrient transporter. GLUT4 is the primary glucose transport protein expressed in skeletal muscle, and animal and human data exist suggesting that endurance training increases the protein levels of this target (Consitt et al., 2019; Evans et al., 2019). Notwithstanding, the available data are limited regarding how RT affects this marker in previously resistance-trained individuals. Studies examining diabetic and pre-diabetic individuals have shown RT significantly increases in GLUT4 protein expression (Holten et al., 2004; Stuart et al., 2017). However, no changes were observed in this marker following VAR and CON protocols. It is possible that untrained diabetics may experience greater RT-induced increases in GLUT4 content compared to healthy, trained individuals (Holten et al., 2004). Additionally, the changes in GLUT4 content may be greater at the onset of training (Langfort et al., 2003).

### Mitochondrial Enzyme Markers

We assayed three mitochondrial enzyme markers involved with substrate oxidation (IDH2, CPT1, and VLCAD) as well as the five protein complexes of the electron transport chain. Moreover, we examined CS activity levels, which is a surrogate marker of mitochondrial volume as discussed prior. IDH2 is an enzyme that facilitates the oxidative decarboxylation of isocitrate within the TCA cycle. Both RT protocols did not alter IDH2 protein expression. CPT1, which regulates the entry of fatty acids into the mitochondria, is believed to play an important role in the fatty acid oxidation (Park et al., 2016). Again, neither form of training altered CPT1 protein expression. VLCAD catalyzes the first step in beta-oxidation. Interestingly, both RT protocols decreased VLCAD protein levels. To our knowledge, this marker has yet to be measured in skeletal muscle following periods of RT, and these findings in conjunction with increases in HK2, may reflect the oxidative to glycolytic phenotype shift known to occur with RT (regardless of the manipulation of RT variables).

Interestingly, neither form of training affected the expression of the five mitochondrial protein complexes. Additionally, although CS activity levels increased after the CON protocol, these values were not higher when compared to the VAR protocol. Larsen et al. (2012) showed that, at a single biopsy time point, CS activity showed excellent agreement with mitochondrial volume as assessed through TEM (*r* = 0.84, *p* < 0.05). Herein, CS activity was normalized to muscle protein. Thus, if CS activity increases from following a period of exercise training, this implicates an expansion of the mitochondria (i.e., an increase in mitochondrial volume). However, results are complicated with RT given that protein accretion also occurs during muscle hypertrophy. Thus, in the presence of whole-tissue or fCSA increases with RT, we have interpreted no change in CS activity as a proportional expansion of the mitochondria. Given that CON and VAR increased fCSA herein, the CS activity data imply that CON disproportionately increased mitochondrial volume from PRE relative to VAR. This is interesting given that we (Roberts et al., 2018; Haun et al., 2019a) and others (Tesch et al., 1987) have reported RT may promote mitochondrial dilution as evidenced through a decrease in CS activity. Moreover, limited evidence suggests that RT increases CS activity (Tang et al., 2006; Cardinale et al., 2017). It is difficult to reconcile why the current data show that CON increased mitochondrial volume, whereas VAR did not. Moreover, it is difficult to reconcile why the complex I-V protein expression data, which similarly reflects mitochondrial volume (Larsen et al., 2012), show no differences from PRE to CON and VAR. However, this likely speaks to the inability of the CS activity assay to detect sensitive changes in mitochondrial volume. In this regard, we have previously reported that 12 weeks of RT does not affect the protein expression of complex I–V proteins in college-aged men, whereas CS activity levels decreased (Roberts et al., 2018). Our laboratory also has histological data suggesting 10 weeks of RT increases mitochondrial expansion at a greater rate than fCSA expansion, whereas CS activity values in these same participants remained unchanged from pre- to post-training (Ruple et al., 2021). The collective findings suggest that, while the CS activity assay may be a good surrogate to show an increase in oxidative capacity occurs in response to endurance exercise in an untrained population (Coggan et al., 1993), using this marker to detect small changes in mitochondrial volume may possess limitations.

### Contractile Protein Markers

A primary aim of our study was to investigate whether the manipulation of RT variables in resistance-trained young men differentially altered sarcoplasmic and myofibrillar spacing, as well relative abundances of major contractile proteins. We observed a decrease in relative MHC abundances following both RT protocols. We also observed decreases in the intracellular area occupied by myofibrils following VAR, despite muscle fiber hypertrophy similar to CON. Finally, we observed that individuals who started training with a greater intracellular density of myofibrils typically experienced an increase in the intracellular area occupied by the sarcoplasm following VAR. All of these findings indicate that both RT protocols, and in particular the protocol with manipulation of RT variables and higher VL given the histology data, may indeed lead to myofiber hypertrophy *via* a more rapid expansion of non-myofibril components. Along with the findings of Haun et al. (2019b), this is the second time we have observed this phenomenon. Mechanistically explaining these findings is difficult given that we did not obtain time-course data from each leg throughout the study. However, when considering studies that have observed similar observations, certain themes emerge. First, participants in the current study, participants from Haun et al. (2019b), and participants from MacDougall et al. (1982) were all previously-trained. However, several of the well-trained bodybuilder and powerlifters in the study by MacDougall et al. (1982) reported using anabolic steroids, and this confounds interpretations. Second, we are more confident in defending the occurrence of sarcoplasmic expansion in the VAR-trained leg, which experienced the effect of frequent manipulation of RT variables and higher VL compared to the CON leg, given that both the SDS-PAGE and histology data suggested this might have occurred. Collectively, these findings suggest the possibility that, in well-trained individuals, unaccustomed RT styles or higher VL may elicit muscle fiber growth through the expansion of the sarcoplasm.

The distance between actin and myosin filaments needs to remain relatively constant to preserve power stroke mechanics and, ultimately, muscle function (Higuchi and Goldman, 1991). Thus, we presume training-induced sarcoplasmic expansion involves spacing between myofibrils, and not within myofibrils. This hypothesis is loosely supported by work from Sanger et al. (2005) that suggests spacing between myofibrils is not rigid, and instead exhibits a range between 2.0–2.5 μm. However, these data are limited to cell culture work, and inter-myofibril spacing in response to exercise has not been interrogated from biopsied human muscle. Another unexplored area that may be related to training-induced sarcoplasmic expansion involves sarcolemma and/or sarcoplasmic protein adaptations to longer-term training. In this regard, Monti et al. (2021) demonstrated that skinned muscle fibers from bodybuilders have a greater propensity to swell in physiological buffer compared to untrained individuals. The authors explained these findings by suggesting RT may affect the expression of fluid transport proteins on the sarcolemma (e.g., AQP4) and/or increase the expression of sarcoplasmic proteins, which in turn, osmotically, drives more fluid into cells. The former contention is supported by recent animal work demonstrating denervation-induced muscle atrophy directly coincides with a decreased expression of AQP4 (Ishido and Nakamura, 2020), and the latter contention is supported by our prior work where an increase in intracellular non-myofibril spacing coincided with increased sarcoplasmic protein concentrations (Haun et al., 2019a). However, these data are limited, and more research is needed to determine if one or both of these mechanisms are responsible for sarcoplasmic expansion. Finally, research in rodents utilizing three-dimensional scanning electron microscopy suggests myofibrils exist in a lattice-like network rather than parallel structures (Willingham et al., 2020). While speculative, this may also hold true in humans and observations of increased sarcoplasmic spacing may be reflective of an enhanced rate of myofibril matrix remodeling rather than sarcoplasmic expansion. In summary, this area of muscle physiology is in its infancy, and more research still needs to be conducted in order to address the following questions: (i) *what is the purpose of cellular growth through the expansion of non-myofibril components?* (ii) *is this form of myofiber growth transient and related to edema or fluid shifts from the extracellular environment, or rather, does this form of hypertrophy serve to spatially prime muscle cells for the eventual accretion of myofibrils and expansion of non-contractile components?* Time-course studies that compare the manipulation RT variables between legs in trained vs. untrained individuals and examine the aforementioned phenomena will be critical in furthering our knowledge in this area.

### Experimental Considerations

There was a lack of agreement between data obtained from histology vs. data produced from biochemical techniques. Specifically, the results of total sarcoplasmic content and specific proteins (determined by SDS-PAGE and Western Blotting) do not completely align with those obtained by actin-phalloidin staining. Although this is difficult to reconcile, this disagreement may be due to the utilized histological techniques providing a two-dimensional representation of dozens of myofibers, while the biochemical techniques analyzed characteristics of ~20 mg of tissue. Finally, due to logistical constraints, we did not determine leg segmental intracellular and extracellular fluid estimates using bioelectrical impedance. Additionally, we did not lyophilize biopsy specimens, as we have done in the past (Haun et al., 2019a; Vann et al., 2020a), in order to ascertain whether fluid content was altered in response to VAR or CON training. Indeed, these assays would have added additional layers of evidence that VAR training may have caused fluid shifts relative to CON training, and this may be responsible for our histological observations. In this regard, future work implementing these assays along with some of the assays performed herein will provide greater clarity on this issue.

## Conclusion

The frequent manipulation of RT variables, despite inducing muscle fiber hypertrophy similar to progressive RT, promotes differential changes in sarcoplasmic and myofibril proteins and a decrease myofibril spacing in resistance-trained individuals. Given that this is only a handful of studies that has reported this phenomenon, more research is needed in this area to underscore the significance of this mechanism.

## Data Availability Statement

The raw data supporting the conclusions of this article will be made available by the authors, without undue reservation.

## Ethics Statement

The studies involving human participants were reviewed and approved by The Human Research Ethics Committee of the Federal University of Sao Carlos. The patients/participants provided their written informed consent to participate in this study.

## Author Contributions

Training and muscle collection occurred in the laboratory of CL. CL and CU designed the study. All wet laboratory analysis occurred at Auburn University in the laboratory of MR. CF, MR, CL, and CU primarily drafted the manuscript. All other co-authors assisted with training or wet laboratory techniques and provided critical insight in the construction of the manuscript. All authors contributed to the article and approved the submitted version.

## Funding

This work was supported by the São Paulo Research Foundation (Grant #2017/05331-6 to VA Grant #2016/24259-1 and #2018/13064-0 to FD Grant #2017/04299-1 and #2020/13613-4 to CL). CL and CU also were supported by the National Council for Scientific and Technological Development (Grant #302801/2018-9 to CL and Grant #303085/2015-0 to CU). Funding for the performed assays and article publishing charges were provided through laboratory donations to MR.

## Conflict of Interest

The authors declare that the research was conducted in the absence of any commercial or financial relationships that could be construed as a potential conflict of interest.

## Publisher’s Note

All claims expressed in this article are solely those of the authors and do not necessarily represent those of their affiliated organizations, or those of the publisher, the editors and the reviewers. Any product that may be evaluated in this article, or claim that may be made by its manufacturer, is not guaranteed or endorsed by the publisher.
